# A case of bleeding from maxillary carcinoma embolized from the maxillary and ophthalmic arteries

**DOI:** 10.1186/s42155-020-00167-6

**Published:** 2020-10-02

**Authors:** Takayuki Sanomura, Takashi Norikane, Kengo Fujimoto, Masahiko Kawanishi, Hiroshi Hoshikawa, Yoshihiro Nishiyama

**Affiliations:** 1 Department of Radiology, Faculty of Medicine, Kagawa University, 1750-1 Ikenobe, Miki-cho, Kita-gun, Kagawa 761-0793 Japan; 2 Department of Neurological Surgery, Faculty of Medicine, Kagawa University, 1750-1 Ikenobe, Miki-cho, Kita-gun, Kagawa 761-0793 Japan; 3 Department of Otolaryngology, Head and Neck Surgery, Faculty of Medicine, Kagawa University, 1750-1 Ikenobe, Miki-cho, Kita-gun, Kagawa 761-0793 Japan

**Keywords:** Maxillary carcinoma, Ophthalmic artery, Embolization

## Abstract

**Background:**

The majority of maxillary sinus cancers are advanced at initial detection due to anatomical limitations and lack of early symptoms. In patients with orbital invasion, the feeding arteries of the tumor are often associated with the ophthalmic artery in addition to the maxillary artery. We describe a case of tumor bleeding in a patient with recurrent maxillary carcinoma that was treated with embolization from the maxillary and ophthalmic arteries.

**Case presentation:**

A 70-year-old man was treated for left maxillary carcinoma from the maxillary artery with 6 cycles of selective intra-arterial cisplatin infusion with concomitant radiation therapy. He subsequently had epistaxis and underwent arterial embolization for hemostasis. He had almost no sight by this time. Angiography of the left external carotid artery and internal carotid artery revealed blood supply from the maxillary and ophthalmic arteries. Regarding the maxillary artery, coil embolization was performed after embolization with 300-500 μm Embosphere. On the other hand, for the ophthalmic artery, the 3rd portion, supratrochlear artery and dorsal nasal artery, were selected and embolized with coils. Final angiography revealed disappearance of tumor staining and a residual choroidoretinal blush. There was no bleeding that needed treatment up to 2 months after embolization.

**Conclusions:**

In embolization of the ophthalmic artery, it is necessary to embolize the second and subsequent parts because the arteries associated with visual function branch off from the first part. Even in patients whose visual acuity has been almost lost, like in this case, there is a risk of eye pain from embolization at the proximal end, and distal embolization is necessary.

## Background

The majority of maxillary sinus cancers are advanced at initial detection due to anatomical limitations and lack of early symptoms. Orbital invasion occurs in 60% to 80% of maxillary sinus malignancies (Carrau et al. 1999). In patients with orbital invasion, the feeding arteries of the tumor are often associated with the ophthalmic artery in addition to the maxillary artery. We describe a case of tumor bleeding in a patient with recurrent maxillary carcinoma that was treated with embolization from the maxillary and ophthalmic arteries. In the embolization of the ophthalmic artery, the artery was related to eye function branches from the first portion of the ophthalmic artery, and so the second and subsequent portions were embolized.

## Case presentation

**Fig. 1:**
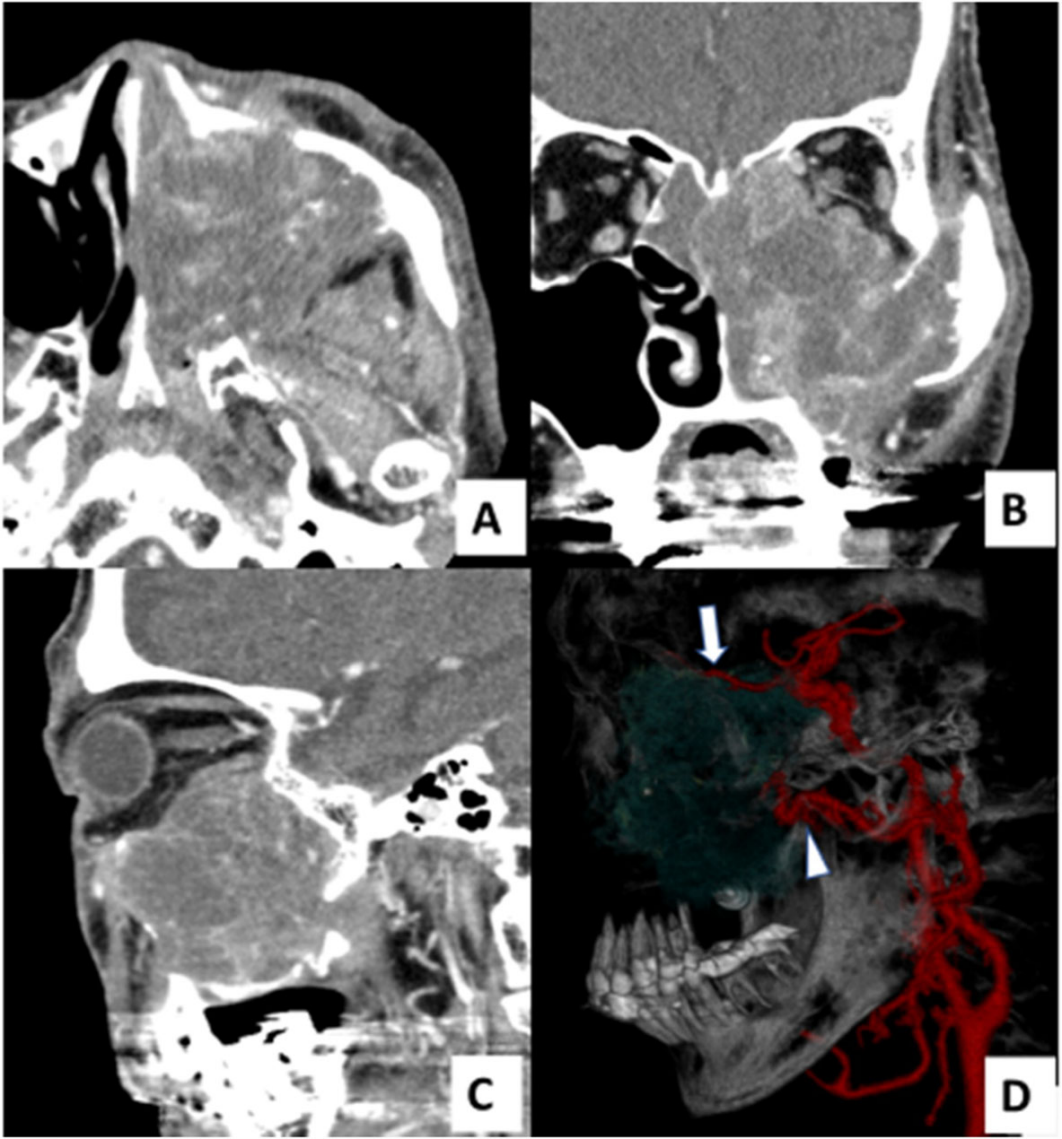
Imaging of recurrent left maxillary carcinoma. A head and neck contrast-enhanced computed tomography showed an irregularly demarcated mass with orbital invasion. **a** Axial image; **b** Coronal image; **c** Sagittal image. **d** Feeding artery was suspected to involve the ophthalmic artery (white arrow) as well as the maxillary artery (white arrowhead)

A 70-year-old man with left maxillary carcinoma (cT4aN0M0, poorly differentiated squamous cell carcinoma) was treated from the maxillary artery with 6 cycles of selective intra-arterial cisplatin infusion with concomitant radiation therapy. However, follow-up CT showed tumor regrowth. The patient subsequently had epistaxis and underwent arterial embolization for hemostasis. A feeding artery was suspected to involve the ophthalmic artery as well as the maxillary artery on contrast-enhanced CT (Fig. 1). He had become almost sightless earlier.

**Fig. 2:**
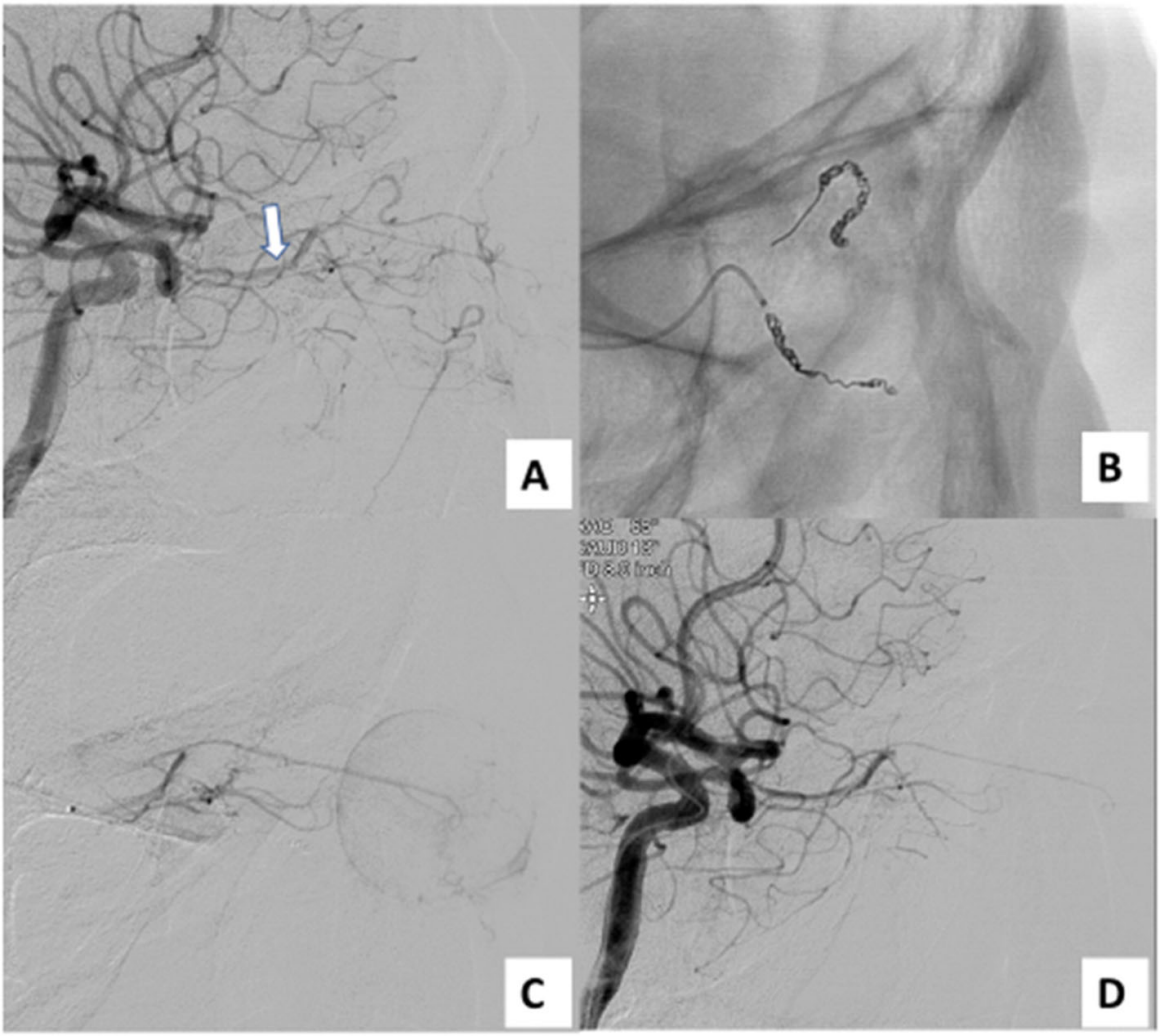
**a** On left internal carotid arteriography the tumor was supplied from the ophthalmic artery (white arrow). **b** The supratrochlear artery and dorsal nasal artery were embolized with coils. **c** Post-embolization angiography revealed disappearance of tumor staining and residual choroidoretinal blush. **d** Post-embolization internal carotid arteriography

**Fig. 3:**
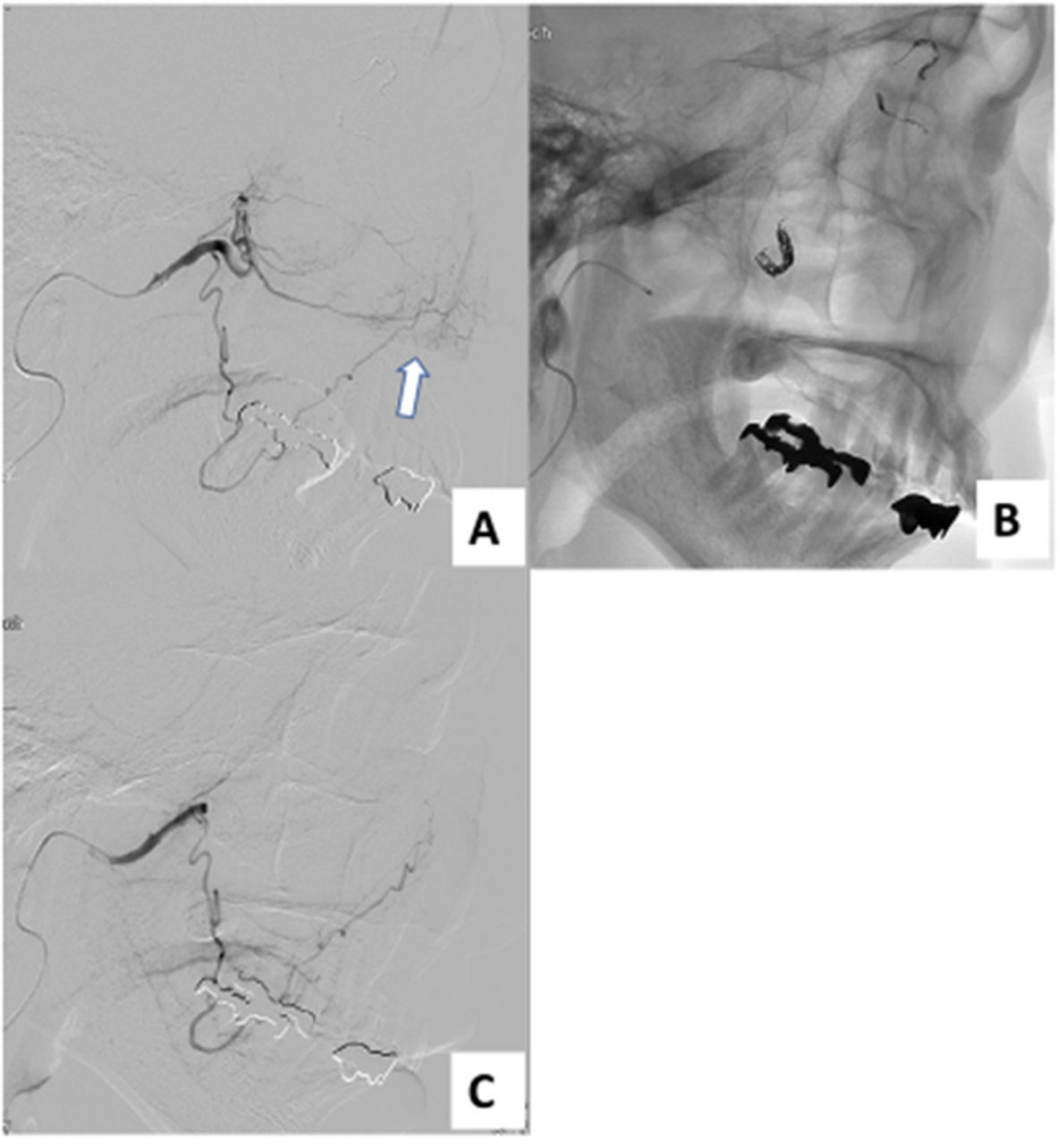
**a** Left maxillary arteriography also showed tumor staining (white arrow). **b** The maxillary artery was embolized with microspheres and coils. **c** Tumor stain disappeared on post-embolization angiography

A 6Fr sheath was placed in the right femoral artery and a 6Fr guiding catheter (Medtronic plc, Minneapolis, MN, USA) was guided to the left common carotid artery. Common carotid arteriography showed the tumor to be supplied from the ophthalmic artery as well as the maxillary artery, as expected from the CT findings. Angiography revealed no extravasation. 2.8Fr/2.6Fr Masters HF (Asahi Intecc, Tokyo, Japan) was guided to the internal carotid artery (Fig. 2), 1.8Fr/1.6Fr Carnelian Marvel S (Tokai Medical Products, Aichi, Japan) was advanced to the ophthalmic artery. Ophthalmic angiography showed that the tumor was supplied from the 3rd portion, supratrochlear artery and dorsal nasal artery. Subsequently, each artery was selected and embolized with Barricade complex SR finishing coils (Balt, Irvine, CA, USA). Post-embolization angiography revealed disappearance of the tumor staining and residual choroidoretinal blush. Regarding the maxillary artery, we decided to perform coil embolization after microsphere embolization (Fig. 3). Masters HF was guided to the trunk of the maxillary artery, Carnelian Marvel S was advanced to the 3rd portion of the maxillary artery. Trisacryl gelatin microspheres (Embosphere®, Nihonkayaku, Tokyo, Japan) 300–500 μm in size mixed with 300 mgI/mL iopamidol (Iopamiron; Bayer, Osaka, Japan) were slowly infused. In total, 1 mL of trisacryl gelatin microspheres mixed with iopamidol were injected step by step. Then, seven microcoils (Target™; Stryker, Fremont, CA, USA) were added to the maxillary artery. No bleeding needed to be treated in the 2 months after embolization.

## Discussion

Head and neck squamous cell carcinoma is the sixth most common cancer worldwide (Sakashita et al. 2014). Malignant tumors of the maxillary sinus account for about 70% of all malignancies of the paranasal sinuses and nasal cavity (Imola and Schramm Jr 2002). The standard treatment for advanced maxillary sinus cancer is radical surgery followed by postoperative radiation therapy. Recently, the superselective intra-arterial infusion of high-dose cisplatin with concomitant radiotherapy has been reported to result in good survival in patients with locally advanced sinonasal cancer at several institutions (Homma et al. 2009; Kanoto et al. 2010).

However, some patients are treatment-resistant, and their outcome is very poor. The majority of patients with maxillary sinus cancer are diagnosed in the locally advanced stage. In previous reports, orbital invasion was noted in 60% to 80% of maxillary sinus malignancies (Carrau et al. 1999).

**Fig. 4:**
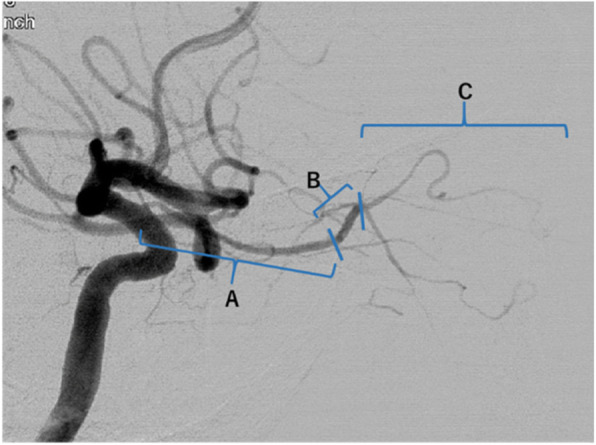
Segmentation of the ophthalmic artery. **a** First portion **b** Second portion **c** Third portion

In the development of the ophthalmic artery, the first horizontally running part arises from the primitive ophthalmic artery (first portion), the part that is slightly ascending and straddles the optic nerve is the anastomosis part (second portion), and its periphery is from the orbital artery (third portion) (Matsumaru et al. 1997; Sato et al. 2019) (Fig. 4). Since the blood vessels related to visual function originate from the primitive ophthalmic artery, the blood vessels do not exist in the periphery of the anastomosis. Therefore, embolization from the ophthalmic artery is safe unless it flows backward beyond the anastomosis.

On the other hand, embolization from the external carotid system must be performed with particular caution to preserve eye function. Loon et al. described that on the contrary later developing internal maxillary artery collaterals may result in inadvertent embolic agent migration to the recurrent meningeal artery - orbital branches of middle meningeal artery, anterior deep temporal artery - lacrimal artery, and sphenopalatine artery/distal internal maxillary artery - ethmoidal arteries. Therefore, a thorough understanding of the vascular supply, hemodynamic characteristics, venous outflow patterns, and collaterals is important for safe and effective treatment (Loon et al. 2017).

In embolization of the ophthalmic artery, it is necessary to embolize the second and subsequent parts because the arteries associated with visual functions branch off from the first part. Even in patients whose visual acuity has been almost lost like in this case, there are risks of eye pain, ocular necrosis, and cosmetic problems from embolization at the proximal end, and distal embolization is necessary.

In our case, embolization was performed as palliative therapy for bleeding associated with local progression. With the development of medical care, the long-term prognosis of advanced cancer patients can be expected to improve, and the importance of palliative care to increase. Accurate embolization can relieve symptoms without causing new complications.

## Conclusions

We presented a case of bleeding from maxillary carcinoma embolized from the maxillary artery and ophthalmic artery. In ophthalmic artery embolization, it is necessary to embolize beyond the first portion to maintain visual function and prevent eye pain.

## Data Availability

The data used the study are available from corresponding author on request.

## Abbreviations

CT: Computed tomography
Fr: French catheter scale
MN: State of Minnesota
CA: State of California
